# MICdb3.0: a comprehensive resource of microsatellite repeats from prokaryotic genomes

**DOI:** 10.1093/database/bau005

**Published:** 2014-02-17

**Authors:** Suresh B. Mudunuri, Sujan Patnana, Hampapathalu A. Nagarajaram

**Affiliations:** ^1^Department of Computer Science & Engineering, Grandhi Varalakshmi Venkatarao Institute of Technology, Bhimavaram, Andhra Pradesh 534 207, India, ^2^Training & Delivery Department, TalentSprint Educational Services, IIIT Campus, Hyderabad, Andhra Pradesh 500 032, India and ^3^Laboratory of Computational Biology, Centre for DNA Fingerprinting & Diagnostics, Hyderabad, Andhra Pradesh 500 001, India

## Abstract

The MICdb is a comprehensive relational database of perfect microsatellites extracted from completely sequenced and annotated genomes of bacteria and archaea. The current version MICdb3.0 is an updated and revised version of MICdb2.0. As compared with the previous version MICdb2.0, the current release is significantly improved in terms of much larger coverage of genomes, improved presentation of queried results, user-friendly administration module to manage Simple Sequence Repeat (SSR) data such as addition of new genomes, deletion of obsolete data, etc., and also removal of certain features deemed to be redundant. The new web-interface to the database called Microsatellite Analysis Server (MICAS) version 3.0 has been improved by the addition of powerful high-quality visualization tools to view the query results in the form of pie charts and bar graphs. All the query results and graphs can be exported in different formats so that the users can use them for further analysis. MICAS3.0 is also equipped with a unique genome comparison module using which users can do pair-wise comparison of genomes with regard to their microsatellite distribution. The advanced search module can be used to filter the repeats based on certain criteria such as filtering repeats of a particular motif/repeat size, extracting repeats of coding/non-coding regions, sort repeats, etc. The MICdb database has, therefore, been made portable to be administered by a person with the necessary administrative privileges. The MICdb3.0 database and analysis server can be accessed for free from www.cdfd.org.in/micas.

**Database URL:** http://www.cdfd.org.in/micas

## Introduction

Microsatellites, also known as Simple Sequence Repeats or Short Tandem Repeats, are the tandem repetitions of nucleotide motifs of size 1–6 bp (1). They are ubiquitous in nature and are found in almost all organisms ranging from viruses to humans (2). Microsatellites are distributed throughout the genomes and are found in both coding and non-coding regions (3). These repeats are of interest for many researchers owing to their unique nature, significance and application in various fields. Microsatellite regions more frequently undergo mutations (point mutations as well as change of repeat number) than the other genomic regions (4). Mutations in microsatellites in the coding regions and non-coding regions are known to affect the processes of transcription and translation and have also been implicated in several diseases (5–7). Microsatellites are the most widely used genetic markers and are also applied in various fields such as DNA fingerprinting, linkage analysis, forensics, paternity studies, etc. (8, 9). During the past decade, microsatellites have gained much importance and several studies have been performed to understand their importance in adaptability and evolution of different organisms.

Till date, many organism-specific microsatellite databases (10–18) including MICdb (19) are being used widely by researchers. MICdb is a relational database of perfect microsatellites extracted from known prokaryotic genomes developed by us. MICdb is linked to a graphical interface called Microsatellite Analysis Server (MICAS) using which the database is queried (20). So far MICdb and MICAS have been upgraded two times and recently these were upgraded further to MICdb3.0 and MICAS3.0 by adding new genomes as well as by adding new tools and interfaces for search and analysis. The new database holds microsatellite data extracted from the completely sequenced prokaryotic genomes that are published in NCBI repository. It has to be noted that there might be other genomes that are sequenced but not yet available at NCBI and such genomes do not form part of MICdb database. MICAS3.0 has been developed in such a way that it can be integrated into other genome databases and for this we will provide the necessary assistance. The following sections describe the various enhancements of MICdb3.0 compared with the earlier versions.

## Database Construction

The microsatellites were identified and extracted from the completely sequenced prokaryotic genomes downloaded from the NCBI genome repository (ftp://ftp.ncbi.nlm.nih.gov/genomes/Bacteria) using IMEx (21, 22) with the following parameters (repeat type: perfect; minimum repeat number: mono:6, di: 3, tri: 2, tetra:2, penta:2, hexa:2). Following Saunders *et al.* (23) we extracted the perfect repeats with tract lengths of at least 6 bp. IMEx was chosen, as this performs better than many other available tools for microsatellite identification (24). To incorporate data into MICdb, which was constructed using MySQL (www.mysql.com), the output files of IMEx were parsed using computer programs developed in C & Java. The database is composed of 27 tables.

MICAS3.0, the web-interface to MICdb3.0, provides three different data access modules—‘Browse’ (search by alphabetical order of genomes), ‘Advanced Search’ (search by user criteria) and ‘Pair-wise Comparison of Genomes’ (compare genomes for microsatellite distribution and densities). This server has been developed using HTML and CSS. The server side scripting has been done using PHP and AJAX. Both MICAS and MICdb have been hosted on a Linux Server containing Apache web-server and special care has been taken to ensure the interactivity and user-friendliness of the system.

## Features and Enhancements

MICdb3.0 and MICAS3.0 have been loaded with many useful features that facilitate the users in analysing microsatellites on-the-fly. The following sub-sections describe the various features and enhancements of the new versions of MICAS and MICdb.

### Updated genome repository

The current version of MICdb hosts the microsatellite data of 5043 prokaryotic sequences that include 4772 bacterial (including 2118 plasmid sequences) and 271 archaeal genome sequences. The earlier versions of MICdb contained microsatellite data of few genomes. MICdb1.0 (19) hosted only 83 genomes on a whole, whereas MICdb2.0 hosted data of 487 genomes (178 bacterial genomes + 288 viral genomes + 21 archaeal genomes). The current version MICdb3.0 hosts repeat data of >5000 prokaryotic sequences that can be updated regularly. MICdb3.0, like its previous version, does not host the repeat data of viruses, as a separate and exclusive microsatellite database exists for viral genomes named Viral Microsatellite Database (VMD) (17). The MICdb database can be updated from time to time using the admin module.

### Visualization module

**Figure 1.**
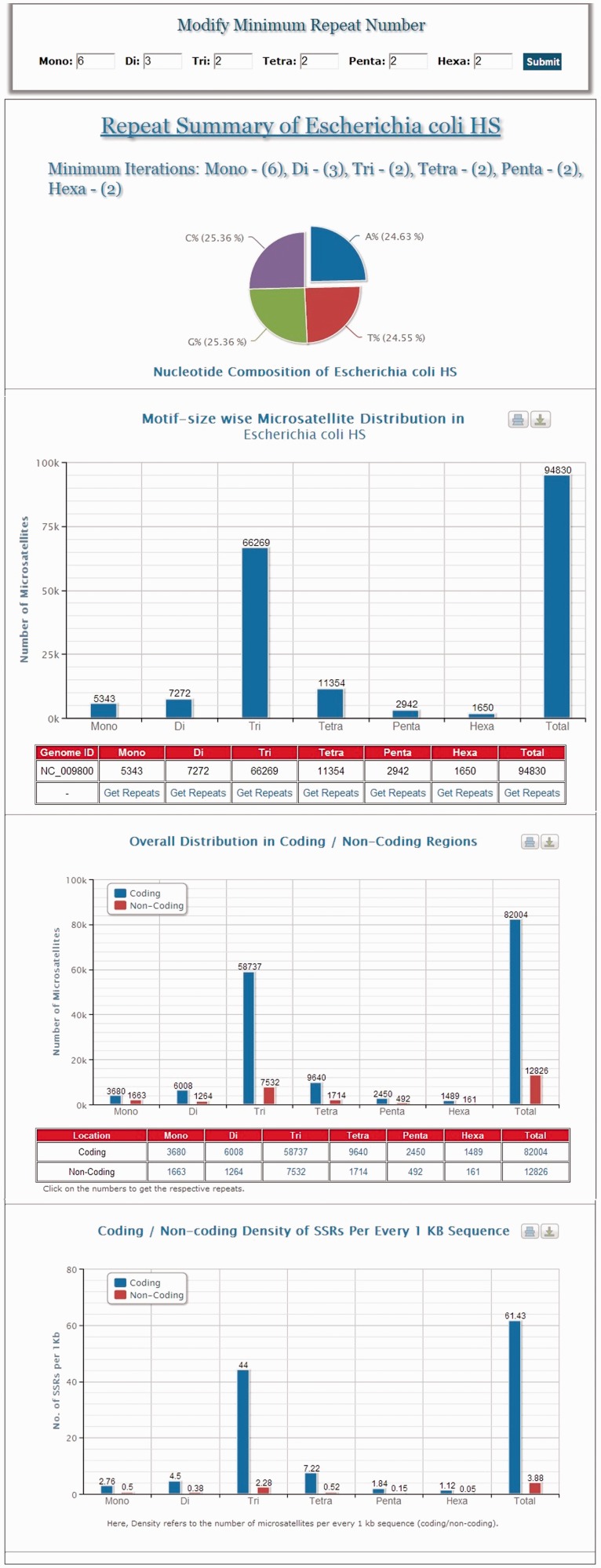
A snapshot of the summary information of *Escherichia coli HS* genome.

MICAS, the web-interface of MICdb, has been powered with a dynamic visualization module that can generate high-quality graphs and charts to depict the distribution and frequencies of various microsatellites found in the queried genomes. The user can get the summary of each genome (Figure 1) in the form of pie and bar charts.

**Figure 2.**
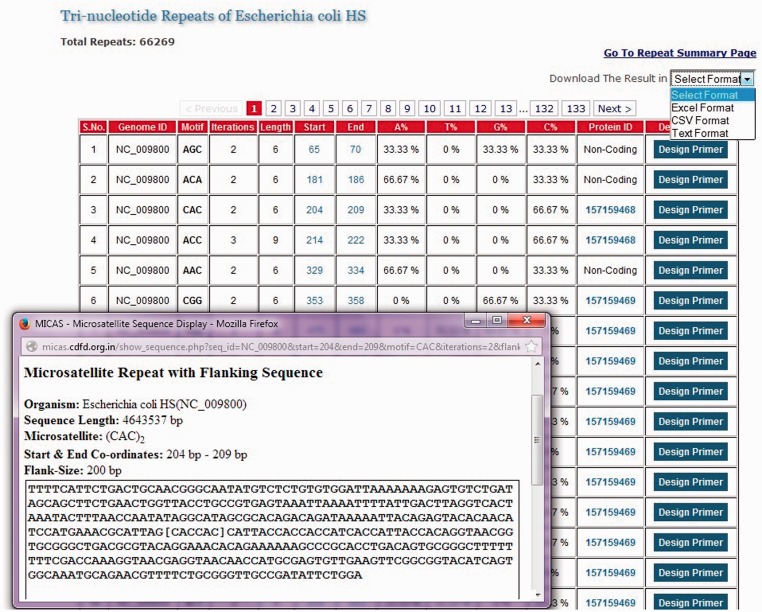
List of all tri-nucleotide microsatellites of *E. coli HS* genome along with a snapshot window of a particular microsatellite repeat along with its summary and flanking sequence.

Using the ‘Browse’ module of MICAS3.0, the users can simply click on a genome of interest (arranged in alphabetical order) and browse through the list of all individual SSRs of that genome and also visualize the motif-wise SSR frequencies and distribution of SSRs in coding and non-coding regions.

The list of SSRs are displayed neatly in tabular format with details such as the repeat motif, iterations, start and end co-ordinates of each SSR, nucleotide composition of each SSR, a link to the protein information if the SSR falls in coding regions and an option to design primer separately for each SSR. Clicking on the co-ordinates will display the complete SSR sequence along with a flanking sequence and summary information of that SSR (Figure 2). An option to export the total SSRs into various formats (.xls, .csv and .txt) has also been provided using which users can download the SSRs and further use them in their analysis.

### Advanced search module

**Figure 3.**
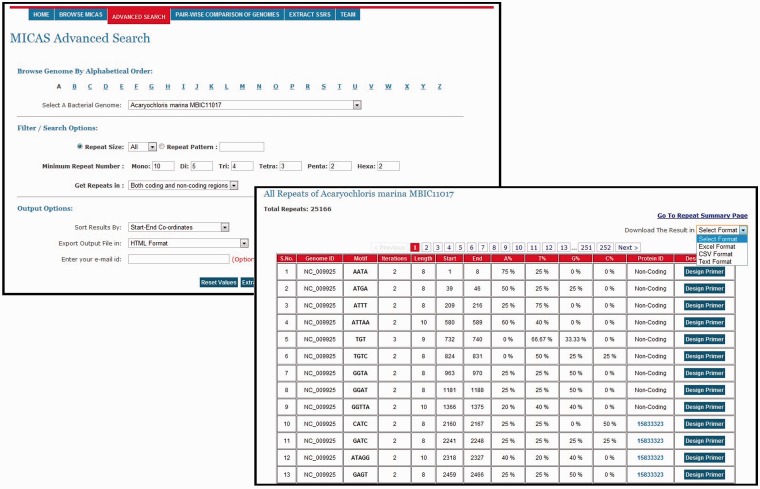
A snapshot of Advanced Search of MICAS and the results page.

To facilitate users for getting repeats based on specific search criteria, MICAS3.0 has been provided with an advanced search module. Using the advanced search module, users can select a particular genome of interest and also specify his/her search criteria and filter repeats accordingly. Advanced search module can filter repeats of a particular size (mono, tri, tetra, etc.,); can get repeats of a particular pattern (CAG, Poly A etc.); can set the minimum repeat number of each motif size; and can filter repeats of only coding or non-coding regions. Moreover, the advanced search module allows you to define the output format (HTML, Excel, CSV or Text) and also sort results based on motif, motif-size or tract length (Figure 3).

### Pair-wise genome comparison

**Figure 4.**
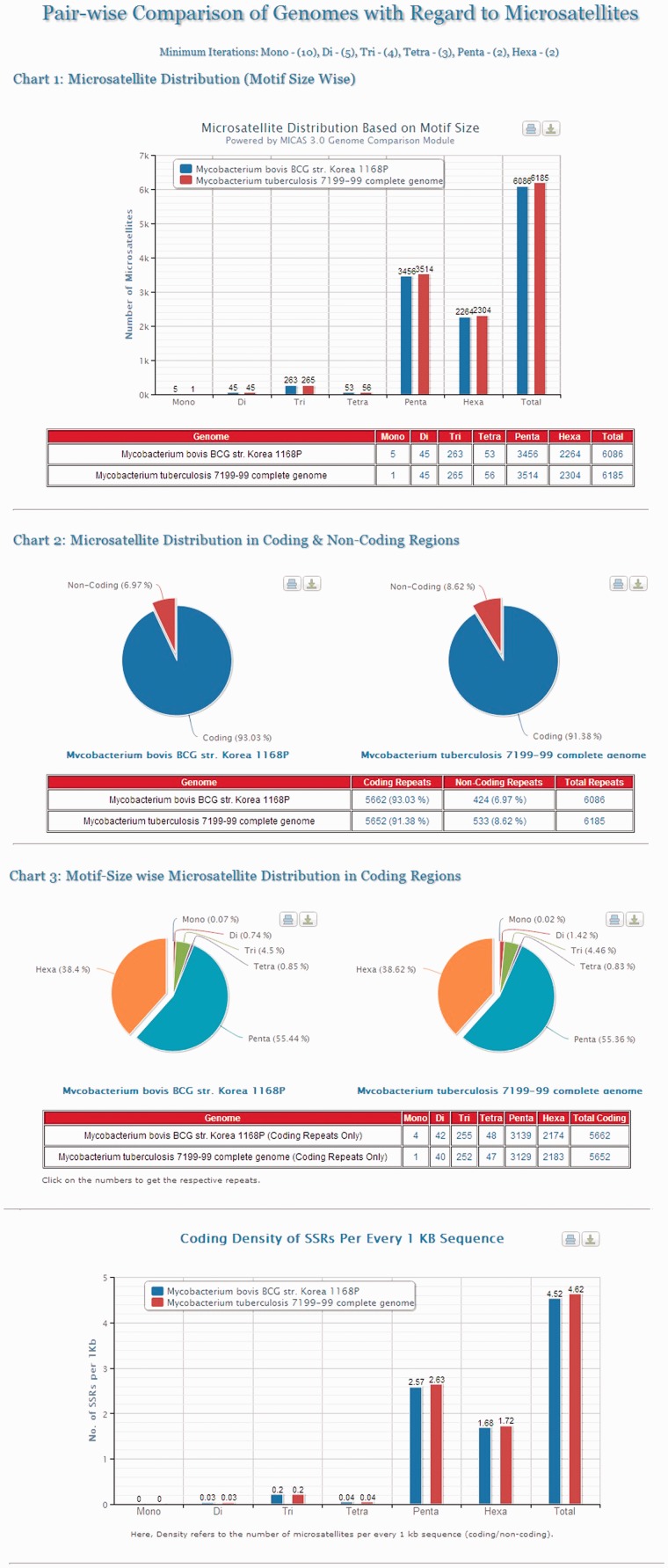
The results page of Pair-wise Genome Comparison of *Mycobacterium tuberculosis 7199-99* complete genome and *Mycobacterium bovis str. Korea 1168P.*

Cross genome comparison is an important area of research to study the genomic evolution of organisms. To aid the evolutionary studies, MICAS is equipped with a unique module for pair-wise comparison of genomes with respect to microsatellite data. Using this module, the users can select any pair of genomes, set their preferred repeat length thresholds and compare the microsatellite distribution in both the genomes. Microsatellite information pertaining to the distribution based on motif-size, distribution in coding/non-coding regions, motif–size-wise distribution and the density of repeats in coding regions of both the genomes can be generated. The results are also displayed neatly in the form of bar/pie charts as well as in tabular formats (Please see Figure 4 for an illustration).

Figure 4 depicts the visualizations of pair-wise genome comparison of distribution of microsatellites of two genomes ‘*Mycobacterium Tuberculosis 7199-99* complete genome’ and ‘*Mycobacterium bovis str. Korea 1168P*’. The graphs will be useful for comparing two closely related genomes where one can compare the motif–size-wise distribution of microsatellites of two genomes side by side apart from their distribution in coding and non-coding regions and their coding densities.

### Results export module

Researchers usually extract microsatellite data of a particular genome and use it for further statistical analysis. Hence, an option to download the results in usable formats has been provided. The results of user queries to MICdb can be exported into different formats such as Text, CSV and Excel. The graphs generated by the visualization module of MICAS can also be downloaded in different image formats such as PNG, JPEG, SVG as well as in PDF format. An option to print the output graphs has also been provided.

### Admin module

**Figure 5.**
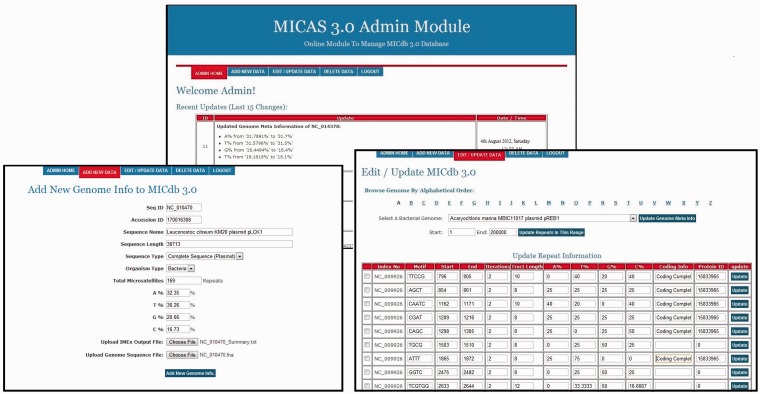
The administration module interface of MICAS3.0 using which the database administrator can add microsatellites of new genomes and can update the database easily*.*

As the number of genomes getting sequenced is increasing rapidly, most of the microsatellite databases are not updated and are outdated. To avoid this problem, MICdb3.0 is equipped with an admin module, a graphical user interface to update the microsatellite data of new genomes from time to time. The MICdb admin needs to login (with a valid user id and password) to the admin module for management of microsatellite and genome data in the database. The admin module can be used to add microsatellite data of new genomes as and when they become available at the NCBI genome repository, edit SSR data of an existing genome as well as delete the unwanted or redundant data from the database. The homepage of admin module displays the list of newly added/modified genomes of NCBI FTP server that are not present in MICdb with update buttons against those genomes. A single click will automatically download the FNA and PTT files of that genome to the MICAS server, submit the files to IMEx for SSR extraction and finally insert the records into the database. It has to be noted that as we use the annotations available at NCBI, they might include errors. Because annotations may also get updated at the NCBI, any such update is identified and the data are updated automatically by the admin module. The Edit feature of admin module has been provided to edit or make corrections to the meta-data and the microsatellite data of a genome. Similarly, the unwanted and redundant data in the database can be deleted directly using the delete option of admin module. A snapshot of the admin module can be found in the Figure 5.

### Primer design module

**Figure 6.**
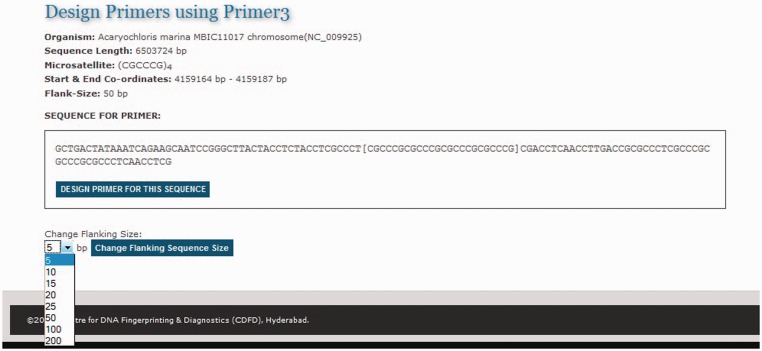
The primer design interface of MICAS3.0 using which the user can prepare the input sequence for primer design with a flanking sequence of his/her choice.

One of the primary needs of researchers studying SSRs is to design primer sequences for specific repeats. To facilitate primer design on the fly MICAS has been connected to the popular primer design software tool Primer3 (25). Each microsatellite repeat that is detected from a genome is hyperlinked to a primer design web interface (Figure 6). The microsatellite repeat along with the left and right flanking sequence is displayed and the sequence can be submitted as input to the primer3 web interface to design primer according to the user choice.

**Figure 7.**
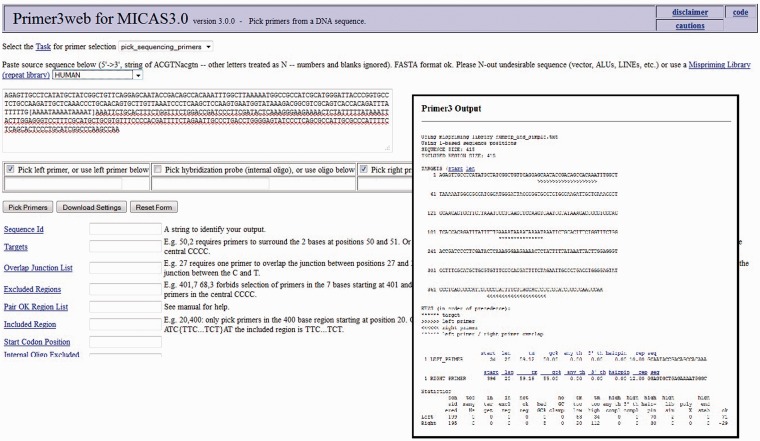
The customized Primer3 interface of MICAS3.0 where the input sequence designed by the user is directly submitted and can be used to modify the various parameters to design primers*.*

After selecting the sequence along with a flanking sequence of user's choice, the page will further be directed to a customized Primer3 web-interface (please see Figure 7) where the sequence is automatically loaded into the input field and user can select various primer designing parameters of primer3 so as to design primers of his/her choice. The flanking sequence size for each microsatellite can be changed dynamically. Instead of storing the flanking sequences in the database, we have included the entire sequence of each genome in a separate table so as to generate the flanking sequences on-the-fly.

## References

[1] Schlotterer C (2000). Evolutionary dynamics of microsatellite DNA. Chromosoma.

[2] Field D, Wills C (1998). Abundant microsatellite polymorphism in Saccharomyces cerevisiae, and the different distributions of microsatellites in eight prokaryotes and *S. cerevisiae*, result from strong mutation pressures and a variety of selective forces. Proc. Natl. Acad. Sci. USA.

[3] Toth G, Gaspari Z, Jurka J (2000). Microsatellites in different eukaryotic genomes: survey and analysis. Genome Res..

[4] Schlotterer C, Ritter R, Harr B (1998). High mutation rate of a long microsatellite allele in *Drosophila melanogaster* provides evidence for allele-specific mutation rates. Mol. Biol. Evol..

[5] Li YC, Korol AB, Fahima T (2004). Microsatellites with in genes: structure, function, and evolution. Mol. Evol..

[6] Gatchel JR, Zoghbi HY (2005). Diseases of unstable repeat expansion: mechanisms and common principles. Nat. Rev. Genet..

[7] Martin P, Makepeace K, Hill SA (2005). Microsatellite instability regulates transcription factor binding and gene expression. Proc. Natl. Acad. Sci. USA.

[8] Wright JM, Bentzen P (1995). Microsatellites: genetic markers for the future. Molecular Genetics in Fisheries.

[9] Goldstein DB, Schlotterer C (2001). Microsatellites: Evolution and Applications.

[10] Sakai T, Miura I, Yamada-Ishibashi S (2004). Update of mouse microsatellite database of japan (MMDBJ). Exp. Anim..

[11] Prasad MD, Muthulakshmi M, Arunkumar KP (2005). SilkSatdb: a microsatellite database of the silkworm, bombyx mori. Nucleic Acids Res..

[12] Blenda A, Scheffler J, Scheffler B (2006). CMD: a cotton microsatellite database resource for gossypium genomics. BMC Genomics.

[13] Gelfand Y, Rodriguez A, Benson G (2007). TRDB-the tandem repeats database. Nucleic Acids Res..

[14] Aishwarya V, Grover A, Sharma PC (2007). EuMicroSatdb: a database for microsatellites in the sequenced genomes of eukaryotes. BMC Genomics.

[15] Archak S, Meduri E, Kumar PS (2007). Insatdb: a microsatellite database of fully sequenced insect genomes. Nucleic Acids Res..

[16] Chang CH, Chang YC, Underwood A (2007). VNTRdb: a bacterial variable number tandem repeat locus database. Nucleic Acids Res..

[17] Mudunuri SB, Rao AA, Pallamsetty S (2009). VMD: Viral Microsatellite Database. A comprehensive resource for all viral microsatellites. J. Comp. Sci. Syst. Biol..

[18] Vouillamoz JF, Arnold C, Frei A (2009). Swiss vitis microsatellite database. Acta Hort. (ISHS).

[19] Sreenu VB, Alevoor V, Nagaraju J (2003). MICdb: database of prokaryotic microsatellites. Nucleic Acids Res..

[20] Sreenu VB, Ranjitkumar G, Swaminathan S (2003). MICAS: a fully automated web server for microsatellite extraction and analysis from prokaryote and viral genomic sequences. Appl. Bioinformatics.

[21] Mudunuri SB, Nagarajaram HA (2007). IMEx: imperfect microsatellite extractor. Bioinformatics.

[22] Mudunuri SB, Kumar P, Rao AA (2010). G-IMEx: a comprehensive software tool for detection of microsatellites from genome sequences. Bioinformation.

[23] Saunders NJ, Jeffries AC, Peden JF (2000). Repeat-associated phase variable genes in the complete genome sequence of Neisseria meningitidis strain MC58. Mol. Microbiol..

[24] Mudunuri SB, Rao AA, Pallamsetty S (2010). Comparative analysis of microsatellite detecting software: a significant variation in results and influence of parameters. Proc. Int. Symp. Biocomput..

[25] Rozen S, Skaletsky HJ, Krawetz S, Misener S (2000). Primer3 on the WWW for general users and for biologist programmers. Bioinformatics Methods and Protocols: Methods in Molecular Biology.

